# 4373 Defining the role of non-canonical PIK3CA mutations in head and neck squamous cell carcinoma

**DOI:** 10.1017/cts.2020.60

**Published:** 2020-07-29

**Authors:** Michelle Ji-Eun Lee, Nan Jin, Janice Cho, Patrick Kwok-shing, Gordon B. Mills, Daniel E. Johnson, Jennifer R. Grandis

**Affiliations:** 1 University Of California, San Francisco

## Abstract

OBJECTIVES/GOALS: To characterize the oncogenic potential of HNSCC cell lines harboring 17 non-canonical *PIK3CA* mutations. METHODS/STUDY POPULATION: Non-canonical *PIK3CA* mutant constructs generated via site-directed mutagenesis are subcloned into doxycycline-inducible vector pLVX-Puro. Serum-dependent HNSCC cell line (PCI-52-SD1) is then stably transfected with vectors and undergo doxycycline-induction. Cell survival is determined by depriving cells of fetal bovine serum for 72 hours and quantifying remaining cells with 3-(4,5-Dimethylthiazol-2-yl)-2,5-diphenyltetrazolium bromide (MTT) assays. Cell proliferation and migration is evaluated with colony formation assays and transwell assays respectively. RESULTS/ANTICIPATED RESULTS: To date, the survival behavior of eight non-canonical mutants was assessed. Three mutants – Q75E, V71I, and E970K – exhibited 18.7-26.7% greater survival rate relative to cells transfected with wild-type. Five mutants – R519G, Y606C, W328S, C905S, and M1040I – demonstrated survival rates that differed only by −4.3% to +6.6% relative to wild-type. We hypothesize the three activating mutants that exhibited increased survival will also demonstrate increased cell proliferation and migratory behavior whereas the three neutral mutants will not differ from control. DISCUSSION/SIGNIFICANCE OF IMPACT: Ongoing HNSCC PI3K inhibitor trials could be more effective if all *PIK3CA* hyperactivation mutations are known. Identifying non-canonical mutation effects could result in greater efficacy if drugs are restricted only to those with activating mutations. CONFLICT OF INTEREST DESCRIPTION: JRG and DEJ are co-inventors of cyclic STAT3 decoy and have financial interests in STAT3 Therapeutics, Inc. STAT3 Therapeutics, Inc. holds an interest in a cyclic STAT3 decoy oligonucleotide. The remaining authors declare no conflicts.

